# The influence of particle size of Enogen Feed corn and conventional yellow dent corn on nursery and finishing pig performance, carcass characteristics and stomach morphology

**DOI:** 10.1093/tas/txab120

**Published:** 2021-07-16

**Authors:** Hadley R Williams, Mike D Tokach, Jason C Woodworth, Joel M DeRouchey, Robert D Goodband, Jordan T Gebhardt, Chad B Paulk

**Affiliations:** 1 Department of Animal Sciences and Industry, College of Agriculture, Kansas State University, Manhattan, KS 66506, USA; 2 Department of Diagnostic Medicine/Pathobiology, College of Veterinary Medicine, Kansas State University, Manhattan, KS 66506, USA; 3 Department of Grain Science and Industry, College of Agriculture, Kansas State University, Manhattan, KS 66506, USA

**Keywords:** α-amylase, Enogen Feed corn, finishing pigs, growth performance, nursery pigs, particle size

## Abstract

Enogen Feed corn is a variety developed by Syngenta Seeds (Downers Grove, IL) that has been genetically modified to contain an α-amylase enzyme trait (SYT-EFC). Originally, Enogen feed corn was developed for the ethanol industry due to its reduction in viscosity of the corn mash, thus eliminating the need to add a liquid form of the α-amylase enzyme. However, there is a potential application for Enogen Feed corn to be used in livestock diets due to the increase in α-amylase enzyme potential to increase starch digestibility. A more common method of increasing starch digestibility in corn is to finely grind it to reduce particle size. This increases the surface area and allows for greater interaction with digestive enzymes. We hypothesized that pigs fed Enogen feed corn potentially could achieve similar gain:feed ratio (G:F) at larger particle sizes than conventional corn because of the differences in starch digestibility. In experiment 1, a total of 360 pigs (DNA 200 × 400, Columbus, NE; initially 6.6 ± 0.1 kg BW) were used with five pigs per pen and 12 pens per treatment. Treatments were arranged in a 2 × 3 factorial with main effects of corn source (Enogen Feed corn or conventional yellow dent corn) and ground corn particle size (300, 600, or 900 µm). Overall, there was a corn source × particle size interaction (linear, *P* = 0.027) for G:F. There was no effect due to particle size when pigs were fed conventional yellow dent corn, but in pigs fed Enogen Feed corn, G:F increased with decreasing particle size. Neither corn source nor particle size affected (*P* > 0.05) overall average daily gain (ADG) or average daily feed intake (ADFI). In experiment 2, a total of 323 pigs (241 × 600; DNA, Columbus, NE; initially 50.0 ± 1.3 kg) were used with nine pigs per pen and six pens per treatment. Treatments were identical as experiement 1. Overall, corn source had no effect on finishing pig ADG, ADFI or G:F. For corn particle size, ADG and G:F increased (linear, *P* < 0.014) and ADFI decreased (*P* = 0.043) as particle size decreased. For stomach morphology, there was a tendency for a corn source × particle size interaction (*P* = 0.055) for keratinization score with keratinization increasing linearly (*P* = 0.001) as particle size of the corn decreased for yellow dent corn with no change in keratinization score as particle size decreased for Enogen Feed corn. In summary, reducing corn particle size improved G:F with no major differences observed between corn sources for overall pig performance.

## INTRODUCTION

Cereal grains such as corn, grain sorghum, wheat, and barley typically provide most of the dietary energy in swine diets. Due to the elevated energy density and availability when compared with other cereal grains, corn is the most commonly used cereal grain among pork production systems in the United States. Corn typically contains 65% starch (NRC, 2012) with apparent total tract digestibility around 90–96% (Rojas and Stein, 2015). Genetic modification of corn varieties has enhanced nutrient profiles. Genetically modified corn varieties, such as NutriDense and low-phytate corn, have been previously evaluated in growing-finishing pigs (Spencer et al., 2000, Hastad et al., 2005). Enogen Feed corn, (Syngenta Seeds, Downers Grove, IL), has been genetically enhanced to contain a thermotolerant α-amylase enzyme trait. While Enogen Feed corn was originally intended to be used by the ethanol industry, the increase in α-amylase may have application in livestock feeding. Recent trial conducted by Ochonski et al. (2019) determined that utilizing Enogen Feed corn in finishing pig diets tended to increase ADG compared to pigs that were fed diets with conventional corn, but there was no influence on feed efficiency.

Although corn varieties can influence growth performance, reducing its particle size can influence performance as well. Grinding corn to reduce particle size increases the surface area, allowing more enzymatic activity to occur and increase the digestibility of both energy and other nutrients (Kim et al., 2002). Research in finishing pigs has shown that reducing corn particle size from 1,000 to 400 µm improves feed efficiency (Wondra et al., 1995). However, decreasing particle size below 700 µm is not always beneficial. Wondra et al. (1995) suggested reducing particle size causes more lesions and greater keratinization of the esophageal region due to the lack of protective mucus in this region of the stomach. We hypothesized that feeding coarse Enogen Feed corn will have the same response as feeding fine particle size conventional corn due to the higher availability of starch. Therefore, the objective of this study was to determine the effects of feeding Enogen Feed corn compared to conventional yellow dent corn at different particle sizes on nursery and finishing pig growth performance, carcass characteristics and stomach morphology.

## MATERIALS AND METHODS

The Kansas State University and Institutional Animal Care and Use Committee approved the protocols used in these experiments.

### Ingredients and Chemical Analysis

All diets for experiments 1 and 2 were manufactured at the Kansas State University O.H. Kruse Feed Technology Innovation Center (Manhattan, KS).

All corn in this study was source from a single source for each corn type. Conventional U.S. No. 2 dent corn and Enogen Feed corn were ground to approximately 300, 600, or 900 µm using a roller mill (Model 924, RMS Roller Grinder, Harrisburg, SD) equipped with 3 roll pairs. The top and middle roll pairs had a speed differential of 1.5 with the front rolls operating at 1,285 rpm and the back rolls at 850 rpm. The bottom roll pair differential was 0.68 with the front roll operating at 1,020 rpm and the back roll at 1,500 rpm. The top, middle, and bottom roll pairs had 2.36 and 2.36 corrugations/cm, 4.72 and 5.51 corrugations/cm, and 6.30 and 7.09 corrugations/cm, respectively. Adjustments were made to the roll gap setting to achieve the desired particle sizes. Samples of ground corn were obtained during each feed manufacturing event for each experiment and analyzed (Table 1; Ward Laboratories, Inc., Kearney, NE) for dry matter (method 935.29; AOAC Inc., 2019), starch (Application #322. 2000), crude protein (method 990.03; AOAC Inc., 2019), ether extract (ANKOM Technology, 2004), acid detergent fiber and neutral detergent fiber (NDF) (ANKOM Technology, 2005), calcium (method 6.3; Kovar, 2003) and phosphorus (method 6.3; Kovar, 2003).

**Table 1.** Chemical analysis of corn varieties of experiments 1 and 2 (as-fed basis)^1^

Item, %	Experiment 1		Experiment 2	
	Conventional^2^	Enogen Feed corn^3^	Conventional	Enogen Feed corn
Dry matter	87.38	87.73	87.72	87.80
Starch	59.68	64.15	60.60	59.76
Crude protein	7.74	7.45	7.68	7.40
Ether extract	4.10	4.10	3.88	4.16
Acid detergent fiber	1.70	1.91	1.55	1.73
Neutral detergent fiber	6.32	7.31	6.20	7.22
Ca	0.10	0.13	0.10	0.11
P	0.23	0.21	0.22	0.21

^1^Corn samples were collected at time of feed manufacturing and pooled for analysis (Ward Laboratories, Inc., Kearney, NE). Each value represents the mean of six analyses per sample.

^2^Yellow dent corn.

^3^Enogen Feed corn, Syngenta Seeds, LLC, Downers Grove, IL.

Particle size analysis was conducted on ground corn samples (100 g) in duplicate according to the ANSI/ASAE S319.2 (1995) standard method. Samples were analyzed both with (5 g) and without a dispersing agent (Gilson Company, Inc., Lewis Center, OH) using two separate stainless-steel sieve stacks (13 sieves each) to prevent residual dispersing agent from affecting results. Both sieve stacks contained agitators and were placed in the Ro-Tap (Model RX-29, W. S. Tyler Industrial Group, Mentor, OH) machine for 15 min, using the same sieve and agitator configurations as Gebhardt et al. (2018).

Representative diet samples from each manufacturing event were obtained from each treatment within experiment and stored at −20°C until analysis. Diet samples were analyzed (Ward Laboratories, Inc., Kearney, NE) for dry matter, crude protein, acid detergent fiber, NDF, Ca, and P using the same procedures as used for ground grain samples.

### Animals and Diets

In both experiments, treatments were arranged in a 2 × 3 factorial with main effects of corn source (conventional yellow dent corn or Enogen Feed corn) and ground corn particle size (300, 600, or 900 µm). Diets were similar between all treatments, and Enogen Feed corn replaced conventional yellow dent corn on an equal weight basis (Tables 2 and 3).

**Table 2.** Diet composition, experiment 1 (as-fed basis)

Item	Phase 1^1^	Phase 2^2^
Ingredient, %		
Corn^3^	56.40	66.45
Soybean meal, 46.5% CP	24.70	29.75
Dried whey	10.00	–
Enzymatically treated soybean meal^4^	5.00	–
Calcium carbonate	0.80	0.80
Monocalcium phosphate, 21% P	0.90	0.90
Sodium chloride	0.50	0.60
L-Lysine HCl	0.45	0.50
DL-Methionine	0.22	0.20
L-Threonine	0.18	0.23
L-Tryptophan	0.03	0.04
L-Valine	0.11	0.13
Trace mineral^5^	0.15	0.15
Vitamin premix^6^	0.25	0.25
Zinc oxide	0.25	–
Phytase^7^	0.04	0.04
Total	100	100
Calculated analysis		
Standardized ileal digestible (SID) amino acids, %		
Lysine	1.35	1.30
Isoleucine:lysine	58	55
Leucine:lysine	116	115
Methionine:lysine	37	36
Methionine and cysteine:lysine	58	58
Threonine:lysine	63	64
Tryptophan:lysine	19.0	19.2
Valine:lysine	70	70
Histidine:lysine	36	37
Total lysine, %	1.49	1.44
NE, kcal/kg^8^	2,445	2,560
SID lysine:NE, g/Mcal	5.52	5.34
Crude protein, %	21.2	20.5
Ca, %	0.71	0.66
P, %	0.62	0.58
Analyzed Ca:analyzed P	1.14	1.13
STTD P, %^9^	0.50	0.45

^1^Phase 1 diets were fed from approximately 6.8 to 11.3 kg.

^2^Phase 2 diets were fed from approximately 11.3 to 24.9 kg.

^3^Enogen Feed corn replaced conventional yellow dent corn on an equal weight basis.

^4^HP300 (Hamlet Protein, Findlay, OH).

^5^Provided per kg of premix: 73g Zn from Zn sulfate; 73 g Fe from iron sulfate; 22 g Mn from manganese oxide; 11 g Cu from copper sulfate; 0.2 g I from calcium iodate; 0.2 g Se from sodium selenite.

^6^Provided per kg of premix: 3,527,399 IU vitamin A; 881,850 IU vitamin D; 17,637 IU vitamin E; 1,764 mg vitamin K; 15.4 mg vitamin B12; 33,069 mg niacin; 11,023 mg pantothenic acid; 3,307 mg riboflavin.

^7^HiPhos 2700 (DSM Nutritional Products, Parisppany, NJ) provided an estimated release of 0.10% STTD P.

^8^NE = net energy.

^9^STTD P = standardized total tract digestible phosphorus.

**Table 3.** Diet composition, experiment 2 (as-fed basis)

Item	Phase 1^1^	Phase 2^2^	Phase 3^3^
Ingredient, %			
Corn^4^	75.45	81.90	85.25
Soybean meal, 46.5 % CP	21.80	15.65	12.35
Calcium carbonate	0.93	0.85	0.85
Monocalcium phosphate, 21% P	0.55	0.40	0.35
Sodium chloride	0.50	0.50	0.50
L-Lysine HCl	0.30	0.30	0.30
DL-Methionine	0.07	0.03	0.02
L-Threonine	0.09	0.10	0.11
L-Tryptophan	0.01	0.02	0.02
Trace mineral	0.15	0.13	0.10
Vitamin premix	0.15	0.13	0.10
Phytase^5^	0.02	0.02	0.02
Total	100	100	100
Calculated analysis			
Standardized ileal digestible (SID) amino acids, %			
Lysine	0.95	0.80	0.72
Isoleucine:lysine	62	61	60
Leucine:lysine	139	148	154
Methionine:lysine	32	31	30
Methionine and cysteine:lysine	58	58	58
Threonine:lysine	63	65	68
Tryptophan:lysine	18.6	18.5	18.7
Valine:lysine	69	70	70
Histidine:lysine	42	43	43
Total lysine, %	1.07	0.90	0.82
Net energy, kcal/kg	2,487	2,529	2,551
SID lysine:net energy, g/Mcal	3.83	3.16	2.82
Crude protein, %	18.4	14.6	13.3
Ca, %	0.60	0.51	0.48
P, %	0.47	0.41	0.38
Analyzed Ca:analyzed P	1.27	1.25	1.26
STTD P, %^6^	0.33	0.28	0.26

^1^Phase 1 diets were fed from d 0 to 14.

^2^Phase 2 diets were fed from d 14 to 42.

^3^Phase 3 diets were fed from d 42 to 83.

^4^Enogen Feed corn replaced conventional corn on an equal weight basis.

^5^HiPhos 2700 (DSM Nutritional Products, Parisppany, NJ) provided an estimated release of 0.10% STTD P.

^6^Standardized total tract digestible phosphorus.

Experiment 1 was conducted at the Kansas State University Segregated Early Weaning facility (Manhattan, KS). A total of 360 barrows (DNA 200 × 400, Columbus, NE; initially 6.6 ± 0.01 kg) were placed in pens with five pigs per pen. Pens (1.22 × 1.22 m) had metal slatted floors and were equipped with a four-hole stainless steel dry feeder and a water cup. Pigs were weaned at approximately 21 d of age and placed in pens based on initial body weight (**BW**), and fed a common pelleted started diet for approximately 7-d. On d 7, which was considered d 0 of the study, pens of pigs were allotted to 1 of 6 dietary treatments in a randomized complete block design with BW as the blocking factor. There were 12 replications (pens) per treatment. Diets were provided ad libitum in mash form and split into two separate phases. Phase 1 was fed from d 0 to 14 and phase 2 was fed from d 14 to 35. Pens of pigs were weighed, and feed disappearance was measured weekly to calculate average daily gain (**ADG**), average daily feed intake (**ADFI**), and gain to feed ratio (**G:F**).

Experiment 2 was conducted at the Kansas State University Swine Teaching and Research Center (Manhattan, KS). A total of 323 pigs (241 × 600; DNA, Columbus, NE; initially 50.0 ± 1.3 kg) were used in an 83-d trial. Pens of pigs were allotted to 1 of 6 dietary treatments in a randomized complete block design with BW as the blocking factor with nine pigs per pen and six pens per treatment. Diets were fed in mash form in three separate phases. The facility was totally enclosed and environmentally regulated, containing 36 pens. Each pen (3.00 × 2.44 m) was equipped with a 1-cup waterer and a dry, single-sided feeder (Farmweld, Teutopolis, IL) with two feeder spaces. Pens were located over a completely slatted concrete floor with a 1.22-m deep pit underneath for manure storage. A robotic feeding system (FeedPro; Feedlogic Corp., Wilmar, MN) was used to deliver and record daily feed additions to each pen. Pens were equipped with adjustable gates to allow space allowance per pig to be maintained if a pig died or was removed during the experiment. Growth performance was assessed by recording BW and feed disappearance every 2 weeks and at the conclusion of the study (d 83).

On d-79, two pigs per pen, one barrow and one gilt of equal weight based on average of the pen weight, were selected and transported to a USDA inspected packing facility (Natural Food Holdings, Sioux Center, IA) for slaughter and to collect stomachs. The stomachs were taken to the Kansas State University Veterinary Diagnostic Laboratory where a scoring system was used to determine the severity of ulceration and keratinization of the esophageal opening of the stomach (DeJong, 2016). Stomach ulceration score was based on a scale of 1 to 4, with 1 = no ulceration, 2 = < 25% ulceration, 3 = 25–75% ulceration and 4 = > 75% ulceration. This scoring criteria was the same for keratinization.

On d 83, the remaining pigs were individually tattooed with a unique ID number, and a radio frequency identification transponder was inserted into the right ear to allow carcass measurements to be recorded on an individual pig basis. Final individual pig weights were taken, and pigs were transported approximately 2.5 h to a commercial packing plant (Triumph Foods, St. Joseph, MO) and held in lairage for approximately 7 h before slaughter. At the plant, hot carcass weight (**HCW**) was determined immediately after evisceration. Backfat and loin depth were measured with an optical probe (Fat-O-Meter, SFK, Herlev, Denmark) inserted between the third and fourth rib (counting from the ham end of the carcass) at a distance approximately 7 cm from the dorsal midline. Percentage lean was calculated using proprietary equations from the packing plant. Carcass yield was calculated by dividing the individual HCW obtained from the packing plant by the individual final live weight obtained at the farm.

### Statistical Analysis

Treatments were analyzed as a randomized complete block design for two-way ANOVA using the *lmer* function from the *lme4* package in R (version 3.5.1 (2018-07-.2)) with pen considered the experimental unit, average pen body weight at allotment to treatment diets as blocking factor included in the model as a random effect, and treatment as fixed effect. The main effects of corn source and particle size, as well as their interactions, were tested. Differences between treatments were considered significant at *P* ≤ 0.05, and a tendency for significance at 0.05 < *P* ≤ 0.10.

## RESULTS

### Chemical and Particle Size Analysis

The chemical analysis for ground corn was similar across sources for both experiments 1 and 2 (Table 1). Samples analyzed with dispersing agent resulted in lower geometric mean diameter (dgw) and greater geometric standard deviation (sgw) than those analyzed without agent as expected (Tables 4 and 5). Kalivoda et al. (2017) observed similar findings when comparing methods and suggested that the inclusion of a dispersing agent best facilitated the movement of fine material through sieves and reduced possible agglomerations due to forces such as static charge. Thus, particle size targets and roller mill settings for both trials were based on the analysis with the inclusion of dispersing agent. For experiment 1, the target particle sizes were achieved and remained similar when comparing conventional to Enogen feed corn (Table 4). For experiment 2, the particle size targets were again achieved and remained similar across corn source with the exception of a lower than expected dgw in phase 1 for the highest particle size for Enogen feed corn (785 µm) when compared to the closer to target conventional corn (911 µm; Table 5).

**Table 4.** Particle size analysis of ground corn, experiment 1^1,2^

Item	Conventional yellow dent corn		Enogen Feed corn	
	With flow agent	Without flow agent	With flow agent	Without flow agent
Particle size phase 1, µm				
300	345	407	345	403
600	576	675	554	564
900	954	1,058	883	972
Particle size phase 2, µm				
300	376	454	328	417
600	606	725	525	646
900	1,069	1,169	926	1,460

^1^Ground corn samples were collected the day of feed manufacturing, values represent the mean of 2 samples.

^2^Ground corn samples were split using a riffle splitter to produce 2, 100 g samples. A dispersing agent (0.5 g of powdered synthetic amorphous silicon dioxide, Gilson Company, Inc., Lewis Center, OH) was added to one of the samples.

**Table 5.** Particle size analysis of ground corn, experiment 2^1,2^

Item	Conventional yellow dent corn		Enogen Feed corn	
	With flow agent	Without flow agent	With flow agent	Without flow agent
Particle size phase 1, µm				
300	343	423	287	419
600	510	673	414	577
900	911	975	785	931
Particle size phase 2, µm				
300	338	434	309	417
600	561	704	567	646
900	932	1,096	983	1,123
Particle size phase 3, µm				
300	374	469	350	476
600	602	743	618	750
900	974	1,167	975	1,202

^1^Ground corn samples were collected the day of feed manufacturing, values represent the mean of 2 samples.

^2^Ground corn samples were split using a riffle splitter to produce 2, 100 g samples. A dispersing agent (0.5 g of powdered synthetic amorphous silicon dioxide) was added to one of the samples.

Complete diet nutrient analysis matched expected values for experiments 1 and 2 (Tables 6 and 7).

**Table 6.** Chemical analysis of diets, experiment 1, (as-fed basis)^1^

Item, %	Phase 1^2^		Phase 2	
	Conventional^3^	Enogen Feed corn^4^	Conventional	Enogen Feed corn
Dry matter	90.32	90.78	89.41	89.42
Crude protein	20.95	20.55	20.35	19.85
Acid detergent fiber	3.10	3.00	3.10	3.30
Neutral detergent fiber	6.50	6.80	5.70	6.60
Ca	0.76	0.81	0.73	0.78
P	0.54	0.52	0.48	0.51

^1^Feed samples were collected approximately 2 days after each feed delivery, pooled within corn source for each phase, and analyzed (Ward Laboratories, Inc., Kearney, NE).

^2^The experimental diets were fed in two phases: d 0 to 14, and d 14 to 35.

^3^Yellow dent corn.

^4^Enogen Feed corn, Syngenta Seeds, LLC, Downers Grove, IL.

**Table 7.** Chemical analysis of diets, experiment 2, (as-fed basis)^1^

Item, %	Phase 1^2^		Phase 2		Phase 3	
	Conventional^3^	Enogen Feed corn^4^	Conventional	Enogen Feed corn	Conventional	Enogen Feed corn
Dry matter	89.15	88.62	88.22	88.53	88.04	88.69
Crude protein	17.25	16.3	14.35	13.65	12.3	12.6
Acid detergent fiber	2.55	2.75	2.65	2.45	2.15	2.35
Neutral detergent fiber	5.55	5.65	4.90	5.75	5.25	5.65
Ca	0.72	0.73	0.76	0.71	0.59	0.59
P	0.42	0.40	0.37	0.34	0.33	0.33

^1^Feed samples were collected approximately 2 days after each feed delivery, pooled within corn source for each phase, and analyzed (Ward Laboratories, Inc., Kearney, NE).

^2^The experimental diets were fed in three phases: d 0 to 14, d 14 to 42, and d 42 to 83.

^3^Yellow dent corn.

^4^Enogen Feed corn, Syngenta Seeds, LLC, Downers Grove, IL.

### Experiment 1

Interactive and main effects are reported in Tables 8 and 9. During phase 1 (d 0 to 14), there was no evidence for interaction or main effect differences for ADG and ADFI for ground corn particle size. However, there was a corn source × particle size interaction (linear, *P* = 0.027) for G:F. There were no changes in G:F for pigs fed decreasing particle size of conventional yellow dent corn, but a linear improvement in G:F was observed as particle size decreased for pigs fed Enogen Feed corn (*P* < 0.001). During phase 2 (d 14 to 35), there was no evidence for differences in ADG among pigs fed either corn source or different particle sizes. There was a tendency for a corn source × particle size interaction (quadratic, *P* = 0.071) for ADFI, with no differences in ADFI for pigs fed conventional yellow dent corn as particle size changed (*P* = 0.540), but an increase then decrease in ADFI as particle size increased in pigs fed Enogen Feed corn. There was also a tendency for a corn source × particle size interaction (linear, *P* = 0.095) for G:F. As particle size of conventional yellow dent corn was reduced from 900 to 600 micron we observed an improvement in G:F and then decreased as the particle size was reduced from 600 to 300 micron. For pigs fed Enogen Feed corn, as particle size was reduced from 900 to 300 micron G:F was improved. There was no difference among pigs fed conventional yellow dent corn, but G:F improved as particle size decreased in pigs fed Enogen Feed corn.

**Table 8.** Effects of corn source and particle size on growth performance of weanling pigs, experiment 1^1^

Item	Conventional, µm^2^			Enogen Feed corn, µm^3^			SEM	Probability, *P* =	
	300	600	900	300	600	900		Source × particle size, linear	Source × particle size, quadratic
BW^4^, kg									
d 0	6.6	6.6	6.6	6.6	6.6	6.6	0.05	0.754	0.856
d 14	11.7	11.7	11.7	12.1	12.3	12.0	0.20	0.911	0.467
d 35	23.9	23.8	23.5	23.9	24.2	23.3	0.33	0.749	0.283
d 0 to 14^5^									
ADG^6^, g	366	363	356	394	405	386	12.3	0.939	0.497
ADFI, g	448	440	440	459	483	482	14.4	0.237	0.472
G:F, g/kg	818	825	810	860	841	800	11.6	0.027	0.945
d 14 to 35									
ADG, g	571	577	562	560	569	540	9.4	0.530	0.584
ADFI, g	907	898	900	882	939	902	19.3	0.408	0.071
G:F, g/kg	631	644	625	636	607	600	9.8	0.095	0.125
d 0 to 35									
ADG, g	489	490	477	494	503	478	8.9	0.801	0.462
ADFI^7^, g	722	712	711	713	757	734	15.3	0.203	0.086
G:F^8^, g/kg	677	689	672	693	666	652	8.0	0.027	0.127

^1^A total of 360 pigs (Line 200 × 400, DNA, Columbus, NE initially 6.6 kg) with 12 pens per treatment and five pigs per pen.

^2^Yellow dent corn.

^3^Enogen Feed corn, Syngenta Seeds, LLC, Downers Grove, IL.

^4^BW = body weight.

^5^The experimental diets were fed in two phases: d 0 to 14, and d 14 to 35.

^6^ADG = average daily gain; ADFI = average daily feed intake; G:F = gain to feed.

^7^Conventional yellow dent corn when particle size of ground corn was reduced (linear, *P* = 0.640), Enogen Feed corn when particle size was reduced (quadratic, *P* = 0.030).

^8^Convetional yellow dent corn when particle size of ground corn was reduced (linear, *P* = 0.610), Enogen Feed corn when particle size was reduced (linear, *P* = <0.001).

**Table 9.** Main effects of corn source and particle size on growth performance of weanling pigs, experiment 1^1^

Item	Source				Particle size, µm				Probability, *P* =	
	Conventional^2^	Enogen Feed corn^3^	SEM	Probability*, P* =	300	600	900	SEM	Linear	Quadratic
BW^4^, kg										
d 0	6.6	6.6	0.05	0.778	6.6	6.6	6.6	0.05	0.531	0.612
d 14	11.7	12.1	0.15	0.001	11.9	12.0	11.8	0.17	0.498	0.394
d 35	23.7	23.8	0.25	0.731	23.9	24.0	23.4	0.27	0.091	0.131
d 0 to 14^5^										
ADG^6^, g	362	395	7.9	0.001	380	384	371	9.2	0.424	0.391
ADFI, g	442	475	10.0	0.003	453	461	461	11.3	0.563	0.712
G:F, g/kg	818	834	6.7	0.094	839	833	805	8.2	0.006	0.268
d 14 to 35										
ADG, g	570	556	6.2	0.063	566	573	551	7.1	0.101	0.064
ADFI, g	902	908	11.4	0.641	895	919	901	15.3	0.708	0.160
G:F, g/kg	633	614	5.9	0.016	634	626	613	6.7	0.036	0.757
d 0 to 35										
ADG, g	485	492	6.3	0.309	491	497	478	7.1	0.090	0.074
ADFI, g	715	734	11.3	0.062	718	734	723	12.5	0.681	0.190
G:F, g/kg	679	671	4.6	0.143	685	678	662	5.7	0.005	0.573

^1^A total of 360 pigs (Line 200 × 400, DNA, Columbus, NE, initially 6.6 kg) with 12 pens per treatment and five pigs per pen.

^2^Yellow dent corn.

^3^Enogen Feed corn, Syngenta Seeds, LLC, Downers Grove, IL.

^4^BW = body weight.

^5^The experimental diets were fed in two phases: d 0 to 14, and d 14 to 35.

^6^ADG = average daily gain; ADFI = average daily feed intake; G:F = gain to feed.

For overall performance (d 0 to 35), there was no evidence for differences in ADG due to corn source or particle size. There was a tendency for a corn source × particle size interaction (quadratic, *P* = 0.086) for ADFI. There was no change in ADFI in pigs fed conventional yellow dent corn regardless of corn particle size, whereas pigs fed Enogen Feed corn had an increase then decrease in ADFI as particle size increased. For overall G:F, there was also a corn source × particle size interaction (linear, *P* = 0.027). There was no difference in G:F when pigs were fed conventional yellow dent corn regardless of corn particle size, but for pigs fed Enogen Feed corn, G:F improved with decreasing particle size.

### Experiment 2

There was no evidence for a corn source × particle size interaction for any growth response criteria (Table 10). There was no evidence for difference in growth performance or carcass characteristics between corn sources, except for a tendency for greater (*P* = 0.064) ADFI from d 56 to 83 of the experiment for pigs fed Enogen Feed corn compared with pigs fed conventional yellow dent corn. For BW, there was a tendency for greater (*P* < 0.10) BW on d 0 and 28 for pigs fed conventional yellow dent corn compared to pigs fed Enogen Feed corn. This small initial difference disappeared by d 56 with no differences in BW between pigs fed either corn source for the remainder of the experiment. As expected, G:F improved (linear, *P* < 0.05) during each phase and overall as corn particle size decreased. Overall, ADFI decreased (linear, *P* = 0.043) as corn particle size decreased (Table 11) while ADG increased (linear, *P* = 0.014) as particle size decreased. The improvement in ADG led to a tendency for increased (linear, *P* = 0.056) BW on d 83 as corn particle decreased.

**Table 10.** Effects of corn source and particle size on growth performance and carcass characteristics of finishing pigs, experiment 2^1^

Item	Conventional, µm^2^			Enogen Feed corn, µm^3^			SEM	Probability, *P* =	
	300	600	900	300	600	900		Source × particle size, linear	Source × particle size, quadratic
BW^4^, kg									
d 0	50.6	50.3	49.8	49.8	48.7	49.7	1.00	0.554	0.228
d 28	78.9	78.6	77.8	78.1	78.3	77.9	0.31	0.314	0.339
d 56	107.1	106.4	104.9	106.6	107.4	105.4	0.63	0.380	0.849
d 83	134.7	132.0	133.1	135.4	134.2	132.1	1.33	0.669	0.600
d 0 to 28									
ADG^5^, g	1,040	1,034	1,006	1,012	1,019	1,004	16.8	0.388	0.883
ADFI, g	2,217	2,250	2,294	2,199	2,323	2,288	41.4	0.731	0.484
G:F, g/kg	468	460	439	460	440	439	6.6	0.681	0.250
d 28 to 56									
ADG, g	1,004	994	967	1,020	1,039	981	21.2	0.960	0.366
ADFI, g	2,886	2,950	2,948	2,946	2,984	3,025	44.7	0.837	0.622
G:F, g/kg	348	337	328	346	348	324	5.2	0.821	0.140
d 56 to 83									
ADG, g	969	939	945	983	973	941	22.9	0.645	0.397
ADFI, g	2,965	2,965	3,079	3,048	3,118	3,090	55.0	0.485	0.248
G:F, g/kg	327	317	308	322	312	305	5.3	0.913	0.915
d 0 to 83									
ADG, g	1,006	990	974	1,006	1,011	976	11.7	0.922	0.327
ADFI, g	2,682	2,714	2,766	2,719	2,799	2,793	34.3	0.957	0.491
G:F, g/kg	375	365	352	370	361	350	3.4	0.895	0.801
Carcass characteristics									
HCW^6^, kg	100.0	98.1	97.8	100.7	99.7	97.6	1.70	0.754	0.600
Carcass yield, %	74.1	74.2	73.6	74.3	74.1	73.8	0.002	0.960	0.259
Backfat depth, mm	16.3	16.3	16.4	16.2	16.4	16.5	0.498	0.871	0.930
Loin depth, mm	67.4	65.2	65.1	66.0	65.1	66.4	0.937	0.171	0.989
Lean, %	54.8	54.4	54.4	54.6	54.4	54.6	0.295	0.519	0.891

^1^A total of 323 mixed gender pigs (Line 241 × 600, DNA, Columbus, NE, initially 50.0 ± 0.3 kg) were used with nine pigs per pen and six pens per treatment.

^2^Conventional yellow dent.

^3^ Enogen Feed corn, Syngenta Seeds, LLC, Downers Grove, IL.

^4^BW = body weight.

^5^ADG = average daily gain; ADFI = average daily feed intake; G:F = gain to feed.

^6^HCW = hot carcass weight.

**Table 11.** Main effects of corn source and particle size on growth performance and carcass characteristics of finishing pigs, experiment 2^1^

Item	Source		SEM	Probability, *P* =	Particle size, µm			SEM	Probability, *P* =	
	Conventional^2^	Enogen Feed corn^3^			300	600	900		Linear	Quadratic
BW^4^, kg										
d 0	50.2	49.4	0.90	0.091	50.2	49.5	49.8	0.93	0.457	0.324
d 28	78.9	77.7	1.04	0.064	78.9	78.1	77.9	1.09	0.164	0.702
d 56	106.6	106.1	1.01	0.494	107.2	106.6	105.1	1.08	0.031	0.564
d 83	133.6	133.5	1.17	0.933	135.4	132.8	132.6	1.32	0.057	0.328
d 0 to 28										
ADG^5^, g	1,028	1,010	10.6	0.185	1,027	1,025	1,005	12.7	0.187	0.497
ADFI, g	2,266	2,260	32.8	0.875	2,220	2,279	2,290	38.2	0.133	0.541
G:F, g/kg	454	448	5.5	0.231	462	451	439	6.2	0.002	0.929
d 28 to 56										
ADG, g	988	1014	13.1	0.128	1,012	1,017	974	15.6	0.067	0.177
ADFI, g	2,929	2,984	25.9	0.131	2,917	2,966	2,987	32.0	0.125	0.702
G:F, g/kg	338	340	3.0	0.615	347	343	326	3.7	0.001	0.166
d 56 to 83										
ADG, g	949	967	13.4	0.349	975	957	943	16.7	0.187	0.932
ADFI, g	3,001	3,086	31.8	0.064	3,004	3,043	3,085	39.4	0.151	0.975
G:F, g/kg	317	313	3.1	0.512	324	315	306	3.9	0.003	0.979
d 0 to 83										
ADG, g	990	998	6.7	0.398	1,005	1,001	975	8.3	0.014	0.289
ADFI, g	2,724	2,767	20.4	0.145	2,701	2,756	2,779	39.4	0.043	0.691
G:F, g/kg	364	360	3.1	0.368	372	363	350	3.9	0.001	0.419
Carcass characteristics										
HCW^6^, kg	98.6	99.3	1.233	0.576	100.3	98.9	97.7	1.355	0.093	0.919
Carcass yield, %	74.0	74.1	0.001	0.437	74.2	74.1	73.7	0.002	0.023	0.253
Backfat depth, mm	16.4	16.3	0.304	0.965	16.5	16.4	16.2	0.357	0.645	0.949
Loin depth, mm	65.9	65.8	0.615	0.921	66.7	65.7	65.2	0.728	0.317	0.193
Lean, %	54.5	54.5	0.175	0.955	54.7	54.4	54.5	0.213	0.509	0.482

^1^A total of 323 mixed gender pigs (Line 241 × 600, DNA, Columbus, NE, initially 50.0 ± 0.3 kg) with nine pigs per pen and six pens per treatment.

^2^Yellow dent corn.

^3^Enogen Feed corn, Syngenta Seeds, LLC, Downers Grove, IL.

^4^BW = body weight.

^5^ADG = average daily gain; ADFI = average daily gain; G:F = gain to feed.

^6^HCW = hot carcass weight.

For carcass characteristics, there was a tendency for an increase (linear, *P* = 0.093) in HCW as corn particle size decreased in the diet. Carcass yield also increased (linear, *P* = 0.023) as corn particle size decreased. For stomach morphology (Table 12), there was a tendency for a corn source × particle size interaction (*P* = 0.055) for keratinization score. This was caused by a lower incidence of keratinization in stomachs of pigs fed Enogen Feed corn when ground to 300 or 900 µm than pigs fed conventional yellow dent corn at these particle sizes with similar keratinization score when both corn sources were ground to 600 µm. Neither corn source nor particle size affected stomach ulceration.

**Table 12.** Effects of particle size on stomach ulceration and keratinization for experiment 2^1^

Item	Conventional^2^			Enogen Feed corn^3^			Probability		
	300	600	900	300	600	900	Source × particle size	Source	Particle size
Ulcer score^4^	1.42	1.42	1.67	1.92	1.50	1.42	0.178	0.438	0.840
Keratinization score	2.92	2.00	1.58	1.42	1.92	1.17	0.055	0.002	0.015

^1^On d 79, two pigs per pen, one barrow and one gilt of equal weight, were selected and transported to Natural Food Holdings, Sioux Center, Iowa to collect stomachs. The stomachs were taken to the Kansas State University diagnostic lab where a scoring system was used to determine the severity of ulceration and keratinization of the esophageal opening of the stomach.

^2^Yellow dent corn.

^3^Enogen Feed corn, Syngenta Seeds, LLC, Downers Grove, IL.

^4^Stomachs were scored on a scale of 1 to 4 with 1 = no ulceration, 2 = <25% ulceration, 3 = 25–75% ulceration and 4 > 75%. This scoring criteria used was the same for keratinization.

## DISCUSSION

The United States is the world’s largest producer of corn, with an annual production of approximately 13 billion bushels in 2019 (NCGA, 2020). Corn in the United States is generally grown for three major uses: food production, livestock use, and the ethanol industry. A new corn hybrid, developed by Syngenta Seeds, Enogen Feed corn, was originally developed with the intent to be used in the ethanol industry. Enogen Feed corn has the ability to reduce the viscosity of its corn mash, thus eliminating the need to add a liquid form of the α-amylase enzyme.

The chemical analysis of ground corn and complete experimental diets found an increase in starch concentration for experiment 1 for ground Enogen Feed corn and an increase in NDF in both the Enogen Feed corn ground corn sample and complete experimental diets. NDF is the general class of polysaccharides that includes most pentosans but do not include certain soluble fibers such as arabino-xylan. NDFs are resistant to digestion by endogenous enzymes and are slowly but incompletely fermented to volatile fatty acids in the large intestine. An increase in dietary NDF decreases energy availability, causing increased feed intake to meet the nutrient requirements for the pig (Sauber and Owens, 2000).

Starch is the major storage carbohydrate of cereal grains. Corn being the most prevalent of cereal grains has a starch content of 65% (NRC, 2012). The digestion of starch is first initiated when feed interacts with salivary amylase (Englyst and Hudson, 2000). Digestion by salivary amylase is short, due to the decrease of pH in the stomach as feed is ingested. Most of the starch digestion takes place in the small intestine, where the starch molecule is hydrolyzed into maltose, maltotriose, and isomaltose subunits by pancreatic α-amylase and isomaltase enzymes (Groff and Gropper, 2000). The digestion of starch is an efficient process, with approximately 95% of starch digestion occurring in the small intestine (Rojas and Stein, 2015). Enogen Feed corn contains an α-amylase enzyme trait (SYT-EFC). The SYT-EFC α-amylase enzyme is thought to help with the starch degradation and improve the digestibility of corn by converting starch into fermentable sugars. Although there is limited research on starch digestion of Enogen Feed corn in monogastrics, a study by Jolly-Breithaupt et al. (2016) observed when finishing cattle were feed corn with the SYT-EFC α-amylase enzyme, there was a numerical increase in post-ruminal starch digestibility compared to cattle fed conventional yellow dent corn.

One of the main drivers of improved feed efficiency is an increase in the ability to utilize energy in the diet (Patience et al., 2015). With the increase of α-amylase in Enogen Feed corn, starch can potentially be converted to sugar more readily, allowing more energy for the animal which in turn should improve feed efficiency. Ochonski et al. (2019) evaluated the use of Enogen Feed corn fed to finishing pigs. While they did not observe any difference in G:F, they observed a numerical increase in ADG when pigs were fed Enogen Feed corn compared to conventional yellow dent corn. In our study, we did not observe any significant differences for ADG, ADFI or G:F when comparing conventional yellow dent corn and Enogen Feed corn on overall finishing pig performance.

Feed processing of cereal grains also can influence feed efficiency. The main reason cereal grains, like corn, are ground to fine particle sizes is to increase particle surface area and energy utilization (Wondra et al., 1995). A study conducted by Rojas and Stein (2015) evaluated the apparent ileal digestibility (AID) of starch and confirmed that decreased corn particle size from 865 to 339 µm linearly increased AID of starch from 89.0% to 96.6%. This is likely due to increased access to the starch granules for α-amylase, which increases starch digestibility in the small intestine. As digestibility increases, feed intake may also decrease because less feed is needed to meet the animal’s nutrient requirements. Although not statistically significant, in both experiments, we observed a numerical decrease in feed intake as corn particle size decreased.

For nursery pigs in experiment 1, as corn particle size was reduced from 900 to 300 µm for Enogen Feed corn, G:F improved linearly. Similarly, Bokelman et al. (2014) also observed improved G:F as diet particle size was reduced. However, a study conducted by DeJong et al. (2014) observed no improvement in G:F when the particle size of corn was reduced from approximately 900 µm to approximately 325 µm for nursery pigs. The reasons DeJong et al. (2014) observed no improvement in G:F was due to decreased ADG and ADFI when the corn was ground to very fine particle sizes. In our study we observed a tendency for a quadratic effect in ADG, with pigs fed 300 and 600 µm corn having increased gain compared to the 900 µm, resulting in a linear improvement in G:F as corn particle size was decreased.

When a pig is weaned, their enzyme activity is relatively low for enzymes like amylase and protease. Amylase is an endogenous enzyme that originates from saliva and brush border enzymes and is used to break down starch in the small intestine. In our study, we observed an improvement in ADG and G:F when pigs were fed Enogen Feed corn compared with pigs fed conventional yellow dent corn the 14 days post weaning. The increase in gain with pigs fed Enogen Feed corn could have been related to the increase in α-amylase in the corn. A study conducted by Yi et al., (2013) found an increase in weight gain when supplementing an enzyme complex of amylase, protease, and xylanase to wean pigs. In our study, the improvement in gain, however, was not maintained during the subsequent period from d 14 to35 after weaning.

Due to a large volume of feed consumed by finishing pigs over their life span, a small improvement in feed efficiency can result in large economic benefits. A study conducted by DeJong et al. (2013) with finishing pigs observed an improvement in G:F when reducing corn particle size from 596 to 320 µm. Other studies have shown that reducing the particle size of corn in the diet can decrease ADFI when fed to finishing pigs (DeJong et al., 2013, Nemechek et al., 2016). In experiment 2, reducing corn particle size from 900 to 300 µm resulted in an increase in ADG, decrease in ADFI, and improvement in G:F. In the current study, a 1% improvement of G:F for every 100 µm decrease of ground corn was observed, which is consistent to the findings by Wondra et al. (1995) who saw an 8% improvement as corn particle size was reduced from 1,000 to 400 µm.

While reducing corn particle size for finishing pigs has been shown to improve G:F, grinding corn to a fine particle has been shown to cause gastric lesions (Maxwell et al., 1970). In experiment 2, there was no evidence for difference in corn source or particle size on ulcer score. However, pigs fed Enogen Feed corn had decreased keratinization score compared to pigs fed conventional yellow dent corn. As corn particle size was decreased from 900 to 300 µm, keratinization score increased for pigs fed conventional yellow dent corn. Keratinization, the process of keratin forming around the esophageal opening of the stomach, occurs prior to ulceration. Once the keratinized tissue is sloughed off, ulcerations are visible. Ulceration occurs with lower particle size due to reduced viscosity of the feed form in the stomach lowering the pH in the stomach. This difference in keratinization scores between corn sources warrants further investigation.

In summary, Enogen Feed corn can be effectively used in nursery or finishing pig diets and result in less keratinization on stomach tissue than pigs fed conventional yellow dent corn. This data also confirms previous research that observed reducing particle size of corn improves G:F in both nursery and finishing pig diets. However, feeding Enogen Feed corn ground to coarser particle size did not yield the same response for G:F as conventional yellow dent corn at finer diet particle sizes as had been hypothesized.
